# Deteriorated Stress Response in Stationary-Phase Yeast: Sir2 and Yap1 Are Essential for Hsf1 Activation by Heat Shock and Oxidative Stress, Respectively

**DOI:** 10.1371/journal.pone.0111505

**Published:** 2014-10-30

**Authors:** Inbal Nussbaum, Esther Weindling, Ritta Jubran, Aviv Cohen, Shoshana Bar-Nun

**Affiliations:** Department of Biochemistry and Molecular Biology, George S. Wise Faculty of Life Sciences, Tel Aviv University, Tel Aviv, Israel; Universidad Autónoma del estado de Morelos, Mexico

## Abstract

Stationary-phase cultures have been used as an important model of aging, a complex process involving multiple pathways and signaling networks. However, the molecular processes underlying stress response of non-dividing cells are poorly understood, although deteriorated stress response is one of the hallmarks of aging. The budding yeast *Saccharomyces cerevisiae* is a valuable model organism to study the genetics of aging, because yeast ages within days and are amenable to genetic manipulations. As a unicellular organism, yeast has evolved robust systems to respond to environmental challenges. This response is orchestrated largely by the conserved transcription factor Hsf1, which in *S. cerevisiae* regulates expression of multiple genes in response to diverse stresses. Here we demonstrate that Hsf1 response to heat shock and oxidative stress deteriorates during yeast transition from exponential growth to stationary-phase, whereas Hsf1 activation by glucose starvation is maintained. Overexpressing Hsf1 does not significantly improve heat shock response, indicating that Hsf1 dwindling is not the major cause for Hsf1 attenuated response in stationary-phase yeast. Rather, factors that participate in Hsf1 activation appear to be compromised. We uncover two factors, Yap1 and Sir2, which discretely function in Hsf1 activation by oxidative stress and heat shock. In Δ*yap1* mutant, Hsf1 does not respond to oxidative stress, while in Δ*sir2* mutant, Hsf1 does not respond to heat shock. Moreover, excess Sir2 mimics the heat shock response. This role of the NAD^+^-dependent Sir2 is supported by our finding that supplementing NAD^+^ precursors improves Hsf1 heat shock response in stationary-phase yeast, especially when combined with expression of excess Sir2. Finally, the combination of excess Hsf1, excess Sir2 and NAD^+^ precursors rejuvenates the heat shock response.

## Introduction

The prevailing and most prominent theories on the process of aging were formulated many years ago, yet the molecular basis and proximal cause(s) of aging remain largely unknown [1]. This gap in knowledge reflects the complexity of longevity, which even in unicellular model organisms such as yeast, involves many and likely interconnected intracellular pathways [2]. The multiple factors and pathways that contribute to lifespan extension are conserved in evolution [3].

The budding yeast *Saccharomyces cerevisiae* emerges as a convenient model organism to study aging at the cellular level because yeast ages within days. Two aging model systems are commonly accepted in *S. cerevisiae*. The replicative lifespan (RLS) is the number of daughter cells a single mother cell can produce. This parameter represents the length of time a single cell remains mitotically active. The chronological lifespan (CLS) is the length of time in which stationary-phase cells remain viable, monitoring the long-term survival of non-dividing, non-mitotic cells [4]. CLS is typically measured by growing yeast culture into the post-diauxic phase that begins ∼24 hrs after inoculation, which is followed by stationary-phase that begins between day 2 and 7 [4]. As yeast undergoes transition from exponential growth to stationary-phase, the thickened cell wall, the decreased metabolism, transcription and translation, and the increased stress resistance, which are characteristic of quiescent cells, are also shared by cells under other conditions. Therefore, the proposed hallmark for quiescent cells is their ability to retain viability and reproduce. Although stationary-phase cultures contain quiescent and nonquiescent cells, which are distinguished by more than 260 transcripts, transcriptional changes occurring during exponential-growth-to-stationary-phase transition are likely common to both quiescent and nonquiescent cells [5]. Hence, chronological aging can be monitored in non-dividing stationary-phase yeast [6].

Not only is *S. cerevisiae* easily amenable to genetic manipulations, but as a unicellular organism facing diverse and ever changing external conditions, yeast has evolved impressive systems that endow them with robust response to such environmental challenges [7]. Regardless of the model organism studied, it is widely accepted that one of the hallmarks of aging is the deteriorating capacity to cope with stresses [8], [9]. The response of *S. cerevisiae* to multiple stresses is largely orchestrated by a single transcription factor, heat shock factor 1 (Hsf1). Hsf1 is conserved from yeast to humans and functions by binding to heat shock element (HSE). The HSE, which was initially discovered in *Drosophila* as an upstream element of the Hsp70 promoter required for heat-induction [10], has now been recognized in the promoter region of Hsf1 target genes in various organisms [11]–[13]. In *S. cerevisiae*, HSE is composed of at least three inverted repeats of the nGAAn/nTTCn module. There is some divergence in the HSEs with respect to number of modules and their spacing, which appears to confer response specificity to the numerous Hsf1-regulated genes [14], [15]. Although Hsf1 binds to HSE as a homotrimer [16], it has been demonstrated in *S. cerevisiae* that HSEs with different architectures are distinctly regulated [17]. The single Hsf1 in *S. cerevisiae*, encoded by the essential *HSF1* gene, is constitutively bound to HSEs, thus maintains basal transcription levels required for viability [16], [18]–[23]. However, heat-induced binding of Hsf1 to specific HSEs has been demonstrated, and genome-wide search for Hsf1 targets has identified global heat-stimulated binding of Hsf1 to multiple target genes. These studies reveal that in response to stress, Hsf1 binding is strengthened and stabilized via Hsf1 hyperphosphorylation and cooperative Hsf1-HSE interactions, resulting in increased transcriptional activity of Hsf1 and enhanced expression of Hsf1 target genes [24]–[28].

Since Hsf1 controls the expression of multiple genes, its response must be regulated yet modular. Modularity in Hsf1 activation can be achieved by the HSE divergence discussed above. Namely, various targets of Hsf1 can respond differently to the same stress, reflecting the Hsf1-HSE mode of interaction, as dictated by the distinct architecture of the HSEs. An additional layer of modularity is the activation of Hsf1 by multiple stresses. Hsf1 responds to heat shock, oxidative stress, glucose starvation, ethanol exposure and osmotic stress [29]–[31]. Hence, to adequately respond to the various stresses, Hsf1 must integrate diverse stimuli. Combining this stress-specific mode of Hsf1 activation with HSEs distinct architectures may lead to fine tuning of the Hsf1 response. Indeed, distinct patterns of Hsf1 hyperphosphorylation have been observed in response to either heat shock or oxidative stress, and kinetic studies suggest differential phosphorylation under different stress conditions [29]. Furthermore, this altered Hsf1 response includes phosphorylation sites that are involved in Hsf1 activation, while other sites contribute to attenuation of Hsf1 activity [20], [32]–[34]. Although the components and signaling pathways that participate in modulating Hsf1 activity remain largely unidentified, it appears that exclusive regulators are involved in activating Hsf1 in response to its various stimuli. For example, the AMP kinase Snf1 is required for Hsf1 activation in response to low glucose but plays no role in Hsf1 activation by heat shock [35].

Hsf1 is considered a pro-longevity gene. The link between stress resistance and longevity is underscored by the fact that genetic and physiological manipulations that extend lifespan in *S. cerevisiae* also confer resistance to various stresses [6]. A genetic screen in *Caenorhabditis elegans* has shown that genes essential for regulating cytoprotective pathways are also required for lifespan extension, establishing the notion that cytoprotection is central to lifespan extension [36]. Hence, if increased stress resistance leads to longevity, Hsf1, which is activated by various stresses, is a plausible candidate for linking stress with longevity. Indeed, in *C. elegans*, overexpression of *HSF1* or its target genes extends lifespan, and Hsf1 is essential for lifespan extension caused by inactivation of the insulin/IGF-1 signaling [37], [38].

Hsf1 is also linked to genes involved in lifespan extension in response to dietary restriction, as Hsf1 in *S. cerevisiae* is activated upon exposure to low glucose [35]. Mechanisms conserved from yeast to humans are implicated in the contribution of dietary restriction to longevity [39]–[41]. Among the genes that are required for this effect are the sirtuins, a family of class III NAD^+^-dependent deacetylases [42]. Whether yeast *SIR2*, the founding member of the sirtuins family, is a pro-longevity gene depends on the yeast aging model system. While *SIR2* is beneficial for RLS, Δ*sir2* cells show prolonged CLS and higher resistance to different stresses [4], [6], [43], [44], including increased aggregation and reduced toxicity of polyglutamine-containing proteins [45]. Also, a functional role of sirtuins in Hsf1 activation was reported in mammals and recently in worms [46], [47].

To address the potential pro-longevity functions of Hsf1, here we examined the activity of Hsf1 during transition of yeast from exponential growth to stationary-phase. The first hint that Hsf1 activity might be compromised came from our study on the aging-dependent aggregation of polyglutamine-containing proteins in yeast. We showed that aggregation of a protein with 47 glutamine residues (47Q) occurred progressively with chronological aging, an effect that was ameliorated by overexpressing Hsf1 [45]. To gain a broader view on the link between aging and Hsf1 functions, here we measure directly Hsf1 activity in response to different stresses during yeast transition from exponential growth to stationary-phase, a scenario representing early stages of chronological aging. Our results show that Hsf1 response to either heat shock or oxidative stress deteriorates in stationary-phase yeast, whereas the response to sugar starvation is maintained. We further show that distinct factors are involved in the activation of Hsf1 by the different stresses; while Yap1 is required for the response to oxidative stress, Sir2 is essential for the heat shock response, and overexpression of Sir2 mimics heat shock. Heat shock response in stationary-phase yeast is restored by a combination of excess *HSF1*, excess *SIR2* and supplementation of NAD^+^ precursor. Taken together with the effect of aging on protein aggregation [45], the results establish *S. cerevisiae* as a suitable model organism not merely for studying lifespan ending in cell death, but also for research addressing the aging process.

## Materials and Methods

### Strains and plasmids

The *S. cerevisiae* wild-type strains used in this study were BY4741 (*MAT*a *his3Δ1 leu2Δ0 met15Δ0 ura3Δ0*) and W303–1b (*MATα ura3-52 trp1Δ2 leu2–3,112 his3–11 ade2–1 can1–100*). Mutants deleted in individual non-essential genes generated from BY4741 [48] included Δ*yap1* and Δ*sir2*. The additional Δ*sir2* mutant (RS1717; W303–1b *sir2*Δ*::his5* ^+^) and a centromeric plasmid for expressing excess *SIR2* (pRS313*-SIR2*; pCLW21) [49] were generously provided by Rolf Sternglanz (Stony Brook University, USA). The library of endogenously expressed GFP-tagged proteins [50] was used to follow two Hsf1 targets harboring different HSEs, Hsp26-GFP (HSE:-888tttttcatttttttatgttttTTCtaGAAccTTCtttacgtgattctcgc-839) and Btn2-GFP (HSE:-365taaagttactgacacttttttTTCtaGAAagTTCcgGAAaattgcgacac-316). The *cdc48-10* temperature-sensitive strain (KFY194; *MATa lys2-801 leu2-3,112 ura3-52 cdc48-10^ts^*) and its wild-type strain (KFY100; *MATa his4-619 leu2-3,112 ura3-52*) were previously described [51]. The HSE2-*lacZ* construct (GA1695; [52]) with a synthetic HSE2 (ctaGAAgcTTCtaGAAgcTTCtagaggatccccg) was generously provided by Ian Dawes (University of New South Wales, Australia). Centromeric plasmids for expressing excess wild-type *HSF1* (pRS314-*HSF1*; pAKS80) or its R206S mutant (pAKS86) were generously provided by Dennis Thiele (Duke University, USA) and Dennis Winge (University of Utah, USA).

### Growth

Yeast used for the β-galactosidase assay were grown in synthetic complete (SC) media containing 2% (w/v) glucose, and yeast used for GFP detection were grown in SC media containing either 2% (w/v) galactose or 2% (w/v) glucose. Drop-out media were used for selecting transformants. Cells were grown at 30°C in 20 ml medium in 100 ml loosely-capped bottles with constant shaking (200 rpm). An overnight culture was inoculated at a specific A_600_ (1 A_600_ = 1.5×10^7^cells/ml), aiming at exponential growth phase (0.2–0.8 A_600_) or stationary-phase (2.0–6.0 A_600_). Where indicated, H_2_O_2_, nicotinamide (NAM; Sigma) or nicotinamide riboside (NR; generously provided by Charles Brenner, University of Iowa, USA) were added to the media at concentrations and timing specified in the figure legends. For glucose starvation experiments, cells were transferred to fresh media supplemented with either standard 2% (w/v) or low 0.05% glucose. For heat shock, cells were exposed for 20 min to 42°C in either water bath or heating block.

### β-galactosidase assay

The assay is based on a protocol described by Guarente et al [53]. Frozen cell pellets were thawed, washed in 1 ml of ice-cold breaking buffer (100 mM Tris-HCl pH 8.0/20% (v/v) glycerol/38.5 mM freshly added β-mercaptoethanol), centrifuged (13,000 rpm; 1 min) and resuspended in screw-capped tubes in 400 µl of ice-cold breaking buffer supplemented with 1.25 mM phenylmethylsulfonyl fluoride (PMSF). Acid-washed glass beads were added, followed by incubation on ice for 30 min. Cells were broken in a mini-bead beater by 2 rounds of 1 min each at maximal speed. Breaking buffer (100 µl) was added and samples were centrifuged (13,000 rpm; 15 min; 4°C). Supernatants were transferred to fresh tubes, and β-galactosidase activity was assayed, as follows: 100 µl of supernatant were added to 900 µl Z-buffer (60 mM Na_2_HPO_4_/40 mM NaH_2_PO_4_/10 mM KCl/1 mM MgSO_4_ (pH 7.0)/38.5 mM freshly added β-mercaptoethanol) and pre-incubated for 10 min at 28°C. After adding 200 µl of *ortho*-nitrophenyl-β-galactoside (ONPG; 4 mg/ml in Z-buffer), yellow color was allowed to develop by further incubation at 28°C. Reaction was stopped by adding 1 M Na2CO3 (500 µl), and absorbance at 420 nm of the *ortho*-nitrophenol (ONP) produced was measured in Genesys 10UV spectrophotometer. Protein concentration was determined with Bradford reagent, using bovine serum albumin as a standard. Specific β-galactosidase activity is calculated as nmol ONP/min/mg protein.

### SDS-PAGE and immunoblotting

The levels of GFP-tagged proteins were estimated by immunoblotting. Equal number of cells (2 A_600_) were lyzed by 30 min incubation on ice in lysis buffer (0.2 M NaOH/0.5% (v/v) β-mercaptoethanol), pH was adjusted to 8.0 with 5 N HCl and samples were boiled for 5 min as previously described [45]. Samples were resolved by SDS-PAGE, transferred to nitrocellulose membranes and probed with rabbit anti-GFP antibody (ab290, Abcam), using mouse anti-actin antibody (ab3280, Abcam) as a loading control. Primary antibodies were followed by DyLight 680-labled goat anti-rabbit IgG (072-06-15-06, KPL) or IRDye 800CW-conjugated goat anti-mouse IgG (LI-COR Biosciences). Secondary antibodies were visualized and quantified by the Odyssey Infrared Imaging System (LI-COR Biosciences).

### Statistics

Experiments with marked differences between treatments were repeated at least 3 times and in most cases 6–9 times. Quantified data are presented as the mean with bars representing the standard errors. Experiments with marginal differences between treatments were repeated more than 10 times and analyzed using SigmaStat software. Statistical tests (paired t-test, Mann-Whitney rank sum test, Kruskal-Wallis one way analysis of variance on ranks) were applied, as detailed in figure legends.

## Results

### Hsf1 response to heat shock deteriorates in stationary-phase yeast

**Figure 1:**
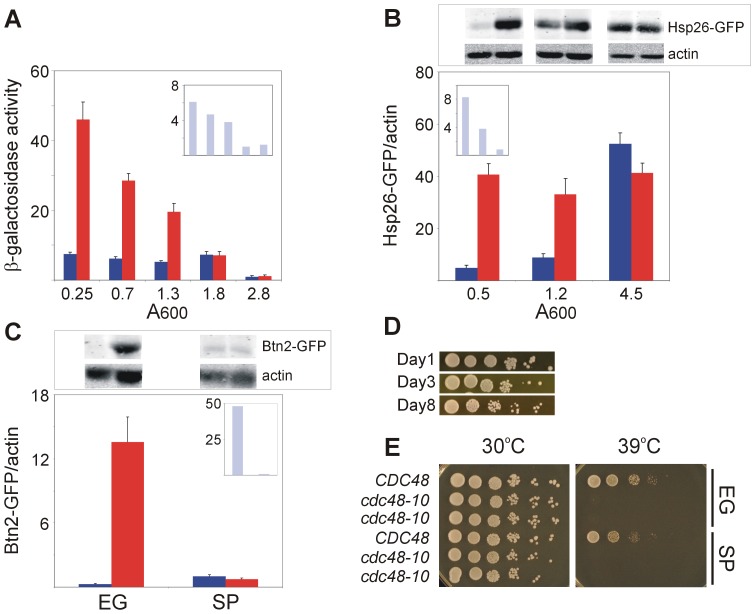
Hsf1 response to heat shock is lost in stationary-phase yeast. BY4741 cells harboring the HSE2-*LacZ* plasmid (A) or expressing Hsp26-GFP (B) or Btn2-GFP (C) were grown at 30°C to the indicated growth stages ((A, B) indicated as A_600_; (C) EG, exponentially-growing; SP stationary-phase). Cells were either incubated for 20 min at 30°C (blue bars) or subjected to a 20 min heat shock at 42°C (red bars). (A) Hsf1 activity was measured as β-galactosidase specific activity. Mann-Whitney rank sum test indicates that the difference between 30°C and 42°C is statistically significant (p<0.001) up to 1.3 A_600_ and not later (A_600_≥1.8). (B, C) Hsf1 activity was measured as levels of Hsp26-GFP or Btn2-GFP relative to actin as a loading control, determined by SDS-PAGE and immunoblotting (upper panels). Blots were visualized and quantified by the Odyssey Infrared Imaging System (LI-COR Biosciences). The data are the mean plus standard error of 7–15 independent experiments. The fold induction by heat shock (A–C, insets) is the ratio of β-galactosidase specific activity or levels of Hsp26-GFD or Btn2-GFP relative to actin, at 42°C and at 30°C. (D) Wild-type BY4741 cells were grown at 30°C and on the indicated days were spotted on rich agar plates as 10-fold serial dilutions starting with 0.5A_600_. (E) Wild-type *CDC48* strain and two independent colonies of the *cdc48-10* temperature-sensitive strain were grown at 30°C either exponentially (EG) or kept in culture for 2 days (SP). Ten-fold serial dilutions starting with 0.5A_600_ were spotted on rich agar plates and incubated for 2 days at either 30°C or 39°C, as indicated.

To gain broad insights into the effect of stationary-phase transition on Hsf1 function, we elicited three different stimuli, heat shock, oxidative stress and glucose starvation, and followed Hsf1 activity using three different reporters: one exogenously introduced, with a synthetic HSE (HSE2-*LacZ*), and two endogenously expressed targets with native HSEs (Hsp26-GFP and Btn2-GFP). These reporters were selected because HSE2-*LacZ* contains 4 inverted repeats of the nGAAn/nTTCn module and is commonly used, 4 and 3 repeats are located in the *BTN2* and *HSP26* promoters, respectively, and both HSEs are of the perfect type [15]. The expression of the HSE2-*LacZ* was followed by measuring β-galactosidase activity (Figure 1A), whereas the levels of the endogenously expressed GFP-tagged Hsp26 and Btn2 [50] were followed by immunoblotting with anti-GFP antibodies (Figure 1B,C). To activate Hsf1 by heat shock, cells were exposed to 42°C for 20 min. Exponentially-growing cells (A_600_<0.8), responded robustly to heat shock, as indicated by the impressive increase in β-galactosidase activity (Figure 1A) or in the levels of Hsp26-GFP (Figure 1B) or Btn2-GFP (Figure 1C). In multiple experiments in exponentially-growing cells, the three Hsf1 reporters responded to heat shock with fold induction ranging from 6 to 50 (Figure 1A–C, insets). However, the response to heat shock progressively declined along the transition (post-diauxic ∼24 hrs culture at 0.8–1.5 A_600_) and it was completely abolished in stationary-phase yeast (day 2–3 at A_600_>2.0) (Figure 1 A–C). These stationary-phase cells were mostly quiescent, since they remained fully viable for up to 3 days under non-dividing conditions (Figure 1D), and their diminished response to heat shock was obvious with the three reporters of Hsf1 activity. Actually, in stationary-phase yeast there were high basal levels of Hsp26-GFP even without heat shock, and exposure to elevated temperature was somewhat inhibitory (Figure 1B). Since neither β-galactosidase nor Btn2-GFP showed high basal levels in stationary-phase yeast, we interpreted this result to reflect mechanisms specific for Hsp26 induction that were independent of Hsf1 but were activated in stationary-phase yeast. Indeed, while Hsf1, as well as the stress-responsive Msn2 and the oxidative stress-responsive Yap1 regulate both Btn2 and Hsp26, transcription of the latter is activated by additional factors, including Gis1, which responds to nutrient depletion, and stress-responsive Msn4 and Cad1, as well as Zap1, Pho2 and Abf1 (SGD project. http://www.yeastgenome.org/download-data).

As stationary-phase cultures are thermotolerant [54], it was important to rule out the remote possibility that such cells are somehow thermally insulated, hence do not sense elevated temperatures. To that end, we tested the effect of high temperature on the growth of the *cdc48-10* temperature-sensitive mutant [51]. Clearly, similar to exponentially-growing cells, two-days old *cdc48-10* mutants failed to grow at 39°C, whereas both exponentially-growing and stationary-phase wild-type cells grew at 39°C, and all cells grew well at 30°C (Figure 1E). We conclude that the inability of stationary-phase yeast to respond to heat shock (Figure 1A–C) is not due to their inability to sense elevated temperature. Rather, it reflects the failure of stationary-phase yeast to activate Hsf1.

### Excess Hsf1 improves Hsf1 activity but hardly restores heat shock response in stationary-phase yeast

**Figure 2:**
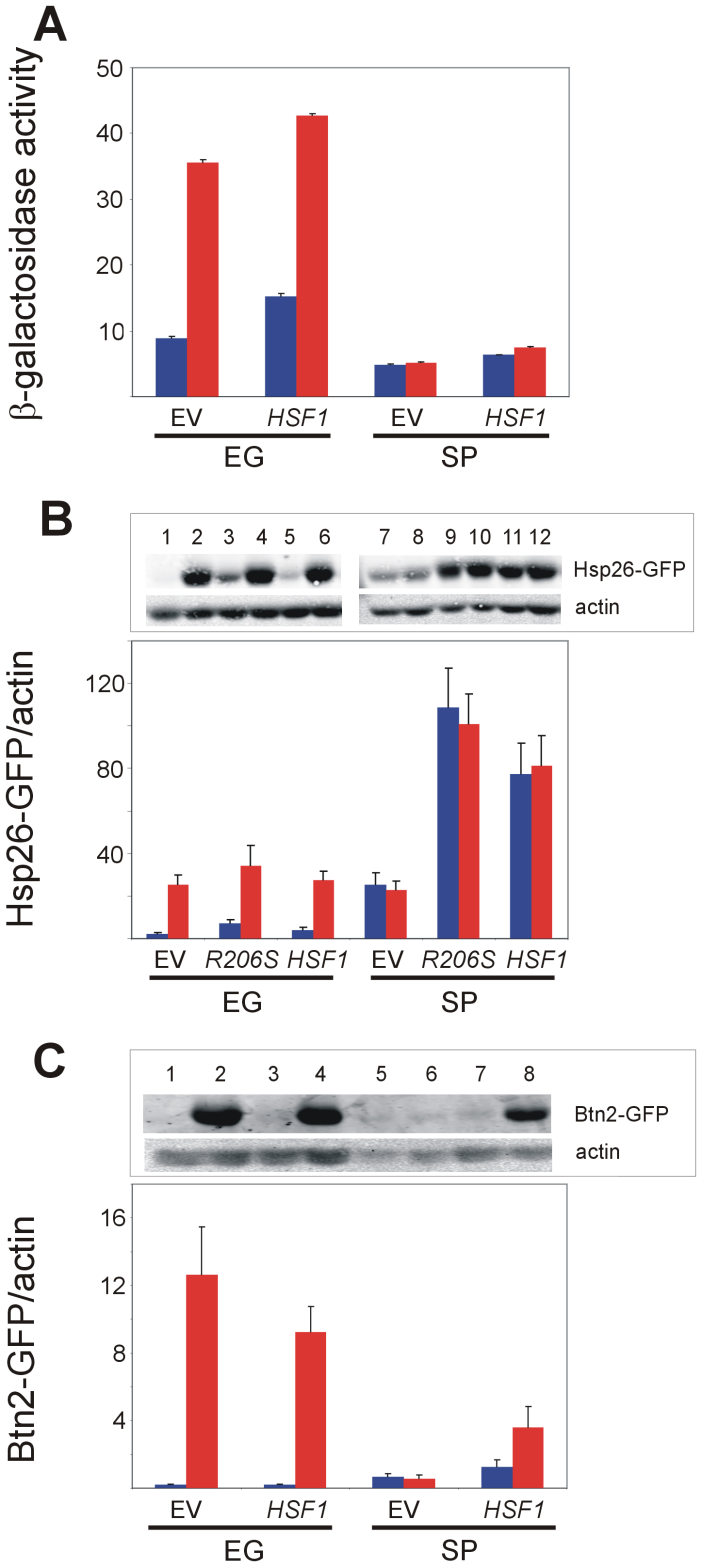
Overexpression of Hsf1 improves its activity in stationary-phase yeast. BY4741 cells expressing HSE2-*LacZ* (A), Hsp26-GFP (B), or Btn2-GFP (C) were transformed with an empty vector (EV) or with centromeric plasmids encoding either wild-type *HSF1* or its *R206S* constitutively-active mutant (only B). Cells grown at 30°C either exponentially (EG) or kept in culture for 2 days (SP). were either incubated for 20 min at 30°C (blue bars) or subjected to a 20 min heat shock at 42°C (red bars). Hsf1 activity in (A) was measured as β-galactosidase specific activity. Hsf1 activity in (B, C) was measured as levels of Hsp26-GFP or Btn2-GFP, respectively, relative to actin (a loading control), as determined by quantified immunoblotting (upper panels). The data are the mean plus standard error of at least 4 independent experiments. Kruskal-Wallis one way analysis of variance on ranks (pairwise multiple comparison with Dunn's method) applied on data in (B) indicates a statistically significant difference between the basal activity in EG cells (p = 0.016) or in stationary-phase cells (p = 0.002) expressing an empty vector (EV) or the *R206S* plasmid, but not between the wild-type *HSF1* and the *R206S* plasmids.

The inability of stationary-phase yeast to respond to heat shock could be the consequence of diminished Hsf1 levels. To test this possibility, we transformed yeast with plasmids to express excess of either wild-type Hsf1 or its constitutively active R206S mutant [55]. Increased basal activity of Hsf1 was observed in exponentially-growing as well as in stationary-phase yeast when excess wild-type Hsf1 was expressed (Figure 2A; 2B, compare lanes 1,5 and lanes 7,11; 2C, compare lanes 5,7). However, excess Hsf1 had a slight effect on the response of stationary-phase yeast to heat shock (Figure 2). This suggests that Hsf1 is somewhat dwindled in stationary-phase yeast but even when overexpressed, other component(s) in the heat shock activation pathway is either missing or becomes limiting. This possibility was corroborated by expressing the constitutively active Hsf1 R206S mutant, which could partially bypass this activation pathway [55]. As shown also here, the R206S mutant could not recapitulate fully the heat shock response, as exponentially-growing yeast still responded to heat shock (Figure 2B, lanes 3, 4). In stationary-phase yeast, the *a priori* higher basal levels of Hsp26-GFP increased further upon expressing the R206S mutant (Figure 2B, lanes 7,9). More importantly, in stationary-phase cells the excess wild-type Hsf1 enabled a weak but discernable heat shock response, reflected by a 1.2-fold increase in β-galactosidase activity (Figure 2A) and in Hsp26-GFP levels (Figure 2B, lanes 11,12) and a 2-fold increase in Btn2-GFP levels (Figure 2C, lanes 7,8). Expression of the R206S mutant abolished the response of stationary-phase yeast to elevated temperature (Figure 2B, lanes 9,10). To conclude, our findings suggest that although the levels of Hsf1 are compromised, the stationary-phase largely impacts on Hsf1 activation. The effects of the constitutively active R206S mutant in exponentially-growing cells was similar to that of the wild-type Hsf1, both still responding to heat shock, whereas in stationary-phase cells the R206S mutant could compensate more efficiently than the wild-type protein for the hampered Hsf1 activation (Figure 2B). This may indicate that R206S cannot bring about its full effect in exponentially-growing cells, where Hsf1 activation is intact and functional. When this activation deteriorates, as in stationary-phase cells, the R206S can partially bypass the need for such activation. This deteriorated Hsf1 activation is indicated by the modest 1.2–2-fold heat shock induction in stationary-phase yeast expressing excess Hsf1, as compared to the substantial 10.7-, 4.1- and 5.1-fold induction upon heat shock in exponentially-growing cells expressing an empty vector, wild-type Hsf1 or R206S, respectively.

### Hsf1 response to glucose starvation is maintained in stationary-phase yeast

**Figure 3:**
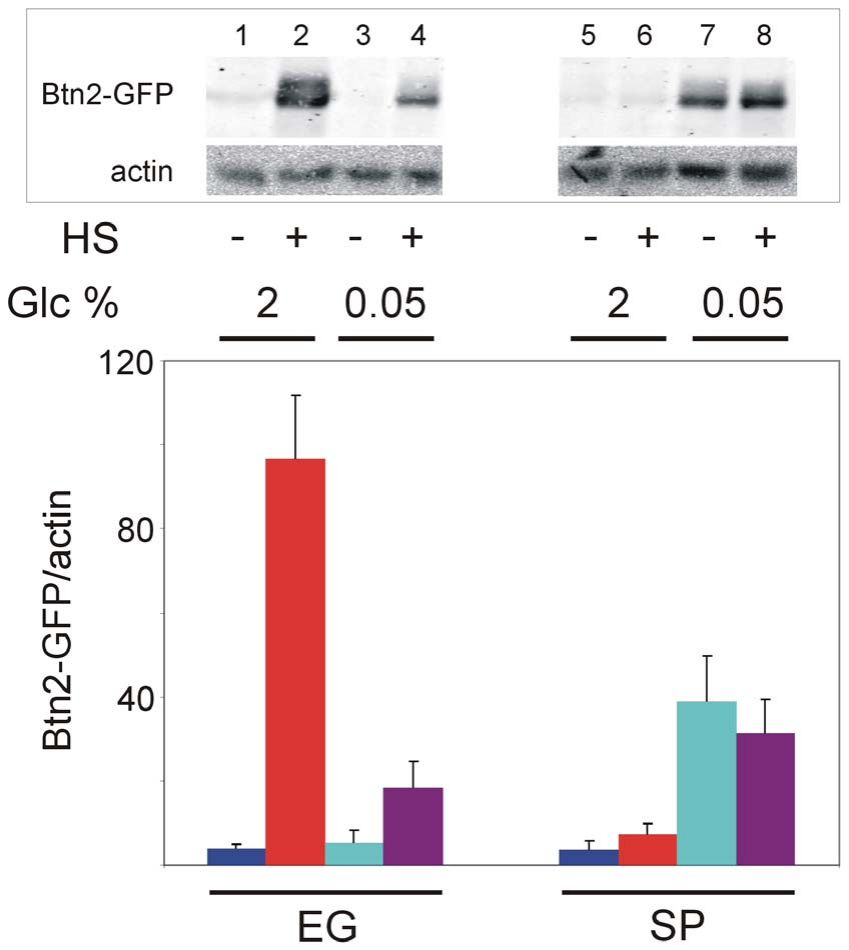
Hsf1 activation by glucose starvation is maintained in stationary-phase yeast. Exponential BY4741 cells expressing Btn2-GFP were grown at 30°C in SC medium containing 2% (w/v) glucose. Cells were transferred to fresh media supplemented with the standard 2% or low 0.05% glucose and were either maintained at exponential growth (EG) or allowed to reach stationary-phase (SP) in these media. Prior to heat shock, cells were transferred to fresh media supplemented with the respective 2% or 0.05% glucose and further incubated at 30°C for 3 hrs. Cells were either incubated for 20 min at 30°C (−) or subjected to a 20 min HS at 42°C (+). Hsf1 activity was measured as levels of Btn2-GFP relative to actin (a loading control), as determined by quantified immunoblotting (upper panel). The data are the mean plus standard error of at least 4 independent experiments.

Since Hsf1 itself did not appear to be a major limiting factor in stationary-phase yeast (Figure 2), we next examined whether the lack of response to heat shock in these cells was manifested also in other modes of Hsf1 activation. In addition to heat shock, Hsf1 is also activated in response to low glucose [8]. For glucose starvation, exponential cells growing at 2% (w/v) glucose were transferred to fresh media supplemented with the standard 2% or low 0.05% glucose and were either maintained at exponential growth or allowed to reach stationary-phase in these media. Prior to heat shock, cells were transferred to fresh media supplemented with the respective 2% or 0.05% glucose. Clearly, stationary-phase yeast, which did not respond to heat shock, maintained their response to glucose starvation by increasing the Btn2-GFP levels when exposed to 0.05% glucose (Figure 3). This response was not accompanied by cell division. Hsf1 responded slightly to glucose starvation in exponential cells but most effectively in stationary-phase cells (Figure 3, lanes 1,3 and 5,7, respectively). Similar results were obtained with Hsp26-GFP at either low glucose or low galactose (Figure S1A), in agreement with the increased Hsp26 mRNA levels in response of exponential yeast to low glucose [35]. Unlike *HSP26* and *BTN2*, the synthetic HSE2-*LacZ* reported poorly on glucose starvation (Figure S1B). Our finding that stationary-phase yeast cannot respond to elevated temperature but maintain their response to low glucose agree with the previously reported independent Hsf1 response to glucose starvation and heat shock [35]. Moreover, it indicates that the inability of Hsf1 in stationary-phase yeast to respond to heat shock at any glucose concentration (Figure 3) is neither due to defects in global protein synthesis nor to considerable depletion of Hsf1 itself. Instead, in stationary-phase cells the heat shock activation of Hsf1 is impaired elsewhere. Although some nutrient depletion was observed, as reflected by improved heat shock response of cells transferred to fresh media, this effect was apparent mostly in exponentially-growing yeast and much less so in stationary-phase cells (Figures 3 and S1). This indicated that the inability of stationary-phase yeast to respond to heat shock was not the consequence of nutrients depletion, since these cells were transferred to fresh media prior to heat shock and still could neither divide nor respond to the elevated temperature (Figure 3, lanes 5–8). Nonetheless, glucose starvation did impose limiting resources for *de novo* Btn2-GFP synthesis, since the intact heat shock response in the exponential yeast was still weaker at low glucose as compared to standard glucose concentration (Figure 3, compare lanes 4 and 2, respectively). Therefore, the ability of stationary-phase yeast to respond to glucose starvation by increasing the Btn2-GFP levels under such limiting conditions was indeed impressive. Yet, such cells did not respond to heat shock (Figure 3, lanes 7,8). We conclude that the inability of stationary-phase yeast to produce β-galactosidase, Hsp26-GFP or Btn2-GFP upon heat shock (Figure 1) reflects an intrinsic modification of their heat shock response, resulting in their failure to activate Hsf1. Since these stationary-phase yeast can still activate Hsf1 by glucose starvation, it rules out the possibility that Hsf1 itself and/or its ability to activate transcription of its target genes is lost in stationary-phase yeast. Instead, it points to factors that participate in Hsf1 activation as components that are compromised.

### Hsf1 response to oxidative stress is lost in stationary-phase yeast and depends on Yap1

**Figure 4:**
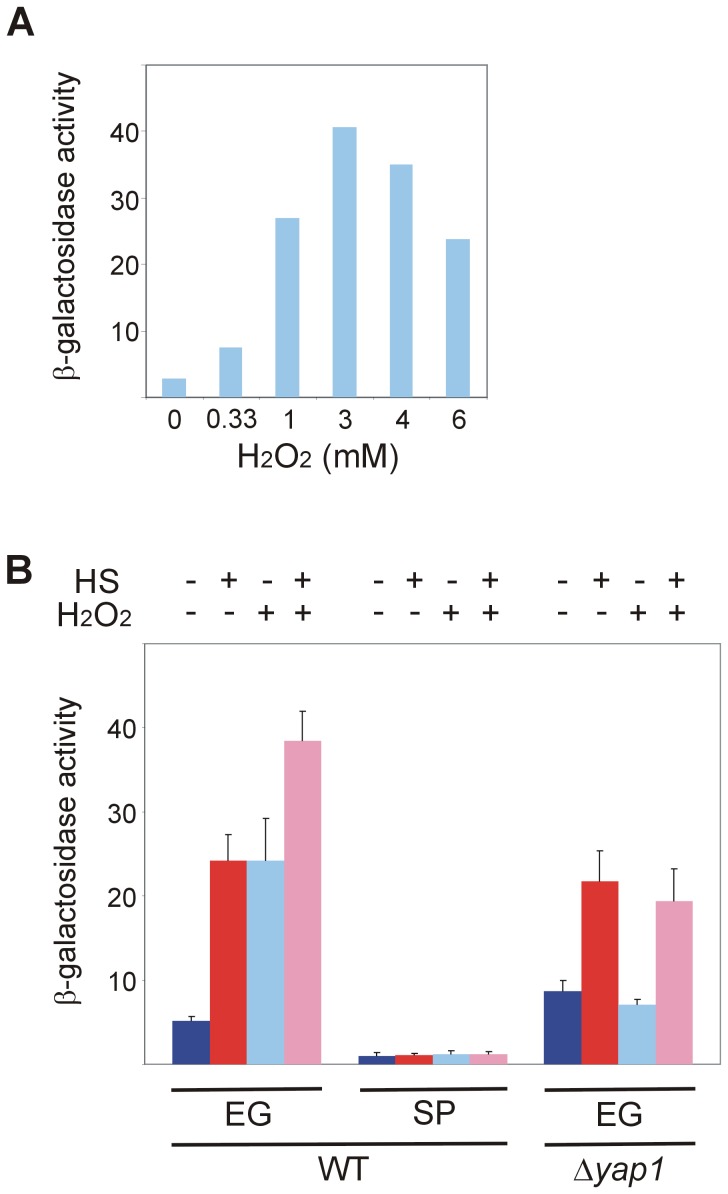
Hsf1 response to oxidative stress is lost in stationary-phase yeast and depends on Yap1. (A) Wild-type BY4741 cells harboring the HSE2-*LacZ* plasmid grown exponentially at 30°C were incubated for 30 min with the indicated concentrations of H_2_O_2_. (B) Wild-type and Δ*yap1* BY4741 cells harboring the HSE2-*LacZ* plasmid grown at 30°C either exponentially (EG) or to stationary-phase (SP) were incubated for 30 min with (+) or without (−) H_2_O_2_ (3 mM) prior to heat shock. Cells were either incubated further for 20 min at 30°C (−) or subjected to a 20 min to heat shock (HS) at 42°C (+). Hsf1 activity was measured as β-galactosidase specific activity. The data are the mean plus standard error of at least 3 independent experiments. Kruskal-Wallis one way analysis of variance on ranks (pairwise multiple comparison with Tukey test) applied on data of EG cells in (B) indicates a statistically significant difference (p<0.001) between the activity in untreated cells and in cells exposed to HS or H_2_O_2_.

Since stationary-phase yeast lost the Hsf1 response to heat shock (Figure 1) but maintained its response to glucose starvation (Figure 3), we tested in these cells the activation of Hsf1 by yet a third stressor, the oxidative stress. Exponentially-growing yeast harboring HSE2-*LacZ* were exposed for 30 min to increasing concentrations of H_2_O_2_ and the measured β-galactosidase activity showed that 3 mM H_2_O_2_ yielded maximal response (Figure 4A), a concentration that was used in subsequent experiments. Notably, the expression of neither Hsp26-GFP nor Btn2-GFP was significantly upregulated by H_2_O_2_ itself, although the heat shock response was augmented in the presence of this oxidant (Figure S2). The β-galactosidase activity revealed that in exponentially-growing yeast H_2_O_2_ activated Hsf1 by itself and the combination of H_2_O_2_ and heat shock generated a stronger activation (Figure 4B). This suggests that oxidative stress and heat shock utilize distinct activation pathways. Conversely, stationary-phase yeast responded neither to heat shock nor to H_2_O_2_ or to their combination (Figure 4B), reflecting deteriorated Hsf1 activation by both stresses.

Next, we examined a potential factor that might play a role in Hsf1 response to oxidative stress. We focused on Yap1, a major transcription factor in the yeast's protective response against oxidative challenges [56]. Yap1 is found in the cytoplasm, and upon exposure to oxidants, this member of the AP-1 family of transcription factors translocates to the nucleus to activate anti-oxidant genes transcription [57]–[62]. Stress regulatory networks studies suggest that Hsf1 and Yap1 operate in parallel pathways, independently activating *PDR3* expression, which leads to *RPN4* and *SNQ2* production [63]. Intrigued by the delayed age-induced cell death upon *YAP1* overexpression [64] and by the notion that Hsf1 and Yap1, two master regulators of stress responses, are also pro-longevity genes [28], we revisited the possible link between Yap1 and Hsf1.

Using the HSE2-*lacZ* reporter, we found that exponentially-growing wild-type cells responded to either heat shock or oxidative stress by augmenting Hsf1 activity, and an even stronger effect was observed in cells challenged with both stresses (Figure 4B). However, while Hsf1 in Δ*yap1* cells was activated by heat shock as efficiently as in wild-type cells, in the absence of *YAP1* the mutant cells no longer responded to oxidative stress, either by itself or in combination with heat shock (Figure 4B). Since Yap1 is not known to directly bind and activate HSEs, these results demonstrate that Yap1 is required to allow activation of Hsf1 by oxidative stress but not by heat shock. Thus, Yap1 must function not only in parallel to, but also upstream of, Hsf1.

### Hsf1 response to heat shock depends on Sir2, is mimicked by excess Sir2, and is improved in stationary-phase yeast by NAD^+^ precursors

The activation of Hsf1 by glucose starvation links Hsf1 to metabolism and possibly to the established lifespan extension by dietary restriction [39]–[41]. In particular, it has been shown that levels of NADPH and NAD^+^ decline upon yeast aging, but NADPH levels are maintained when yeast cells are starved [65]–[67]. Decline in NAD^+^ levels with aging were also reported in mice [68]. Moreover, nicotinamide riboside (NR), which increases NAD^+^ levels [69], [70], extends lifespan [65], [66], directly linking NAD^+^ levels to aging.

**Figure 5:**
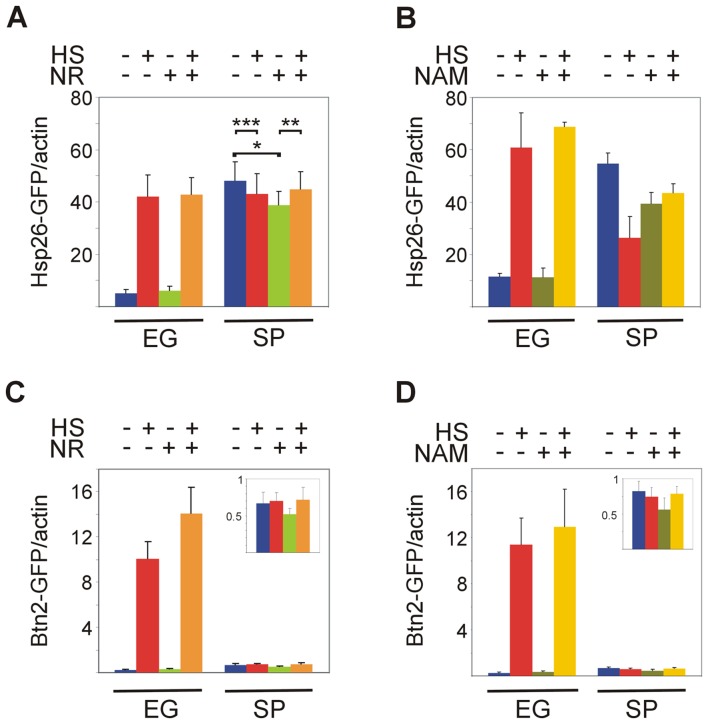
Precursors of NAD^+^ affect Hsf1 activity. BY4741 cells expressing Hsp26-GFP (A,B) or Btn2-GFP (C,D) grown at 30°C either exponentially (EG) or to stationary-phase (SP) were incubated for 30 min with either NR (10 µM; A,C (+)) or NAM (10 mM; B,D (+)) prior to the heat shock. Cells were either incubated further for 20 min at 30°C (−) or subjected to a 20 min to heat shock (HS) at 42°C (+). Hsf1 activity was measured as levels of Hsp26-GFP (A,B) or Btn2-GFP (C,D) relative to actin (a loading control), as determined by quantified immunoblotting. Insets in C, D, levels of proteins in SP yeast drawn to a smaller scale. The data shown are mean plus standard error of at least 10 independent experiments. Kruskal-Wallis one way analysis of variance on ranks (pairwise multiple comparison with Tukey test) applied on data of EG cells in (A) indicates a statistically significant difference (P = 0.001) between untreated cells and cells exposed to either HS or to HS plus NR, and between 30°C and 42°C in cells exposed to NR. Paired t-test applied to data of SP cells in (A) indicates a statistically significant difference (p = 0.07) between untreated cells and cells exposed to NR (*), and between 30°C or 42°C only in cells exposed to NR (**). There is no statistically significant difference between 30°C and 42°C in cells not exposed to NR (***).

To investigate the possible contribution of NAD^+^ to Hsf1 activation, we supplied yeast with NR or nicotinamide (NAM) in order to increase NAD^+^ levels [69], [70]. Addition of NR or NAM to exponentially-growing cells had no significant effect on the basal activity or the heat shock response of Hsf1, as manifested by the levels of Hsp26-GFP or Btn2-GFP (Figure 5). In stationary-phase yeast, NR or NAM also exerted similar responses. On one hand, they attenuated the basal activity of Hsf1, as reflected by the levels of Hsp26-GFP (Figure 5A,B) or Btn2-GFP (Figure 5C,D, insets). More importantly, both NAD^+^ precursors inverted the effect of heat shock. Instead of its inhibitory effect in untreated stationary-phase yeast, supplementing either NR or NAM allowed a slight activation of Hsf1 by heat shock (Figure 5). These marginal effects are statistically significant, showing a difference in the basal Hsf1 activity in stationary-phase yeast between untreated cells and cells supplemented with either NAD^+^ precursor, as well as between 30°C and 42°C only in cells exposed to NAD^+^ precursor, but not in cells not supplemented with NAD^+^ (Figure 5).

**Figure 6:**
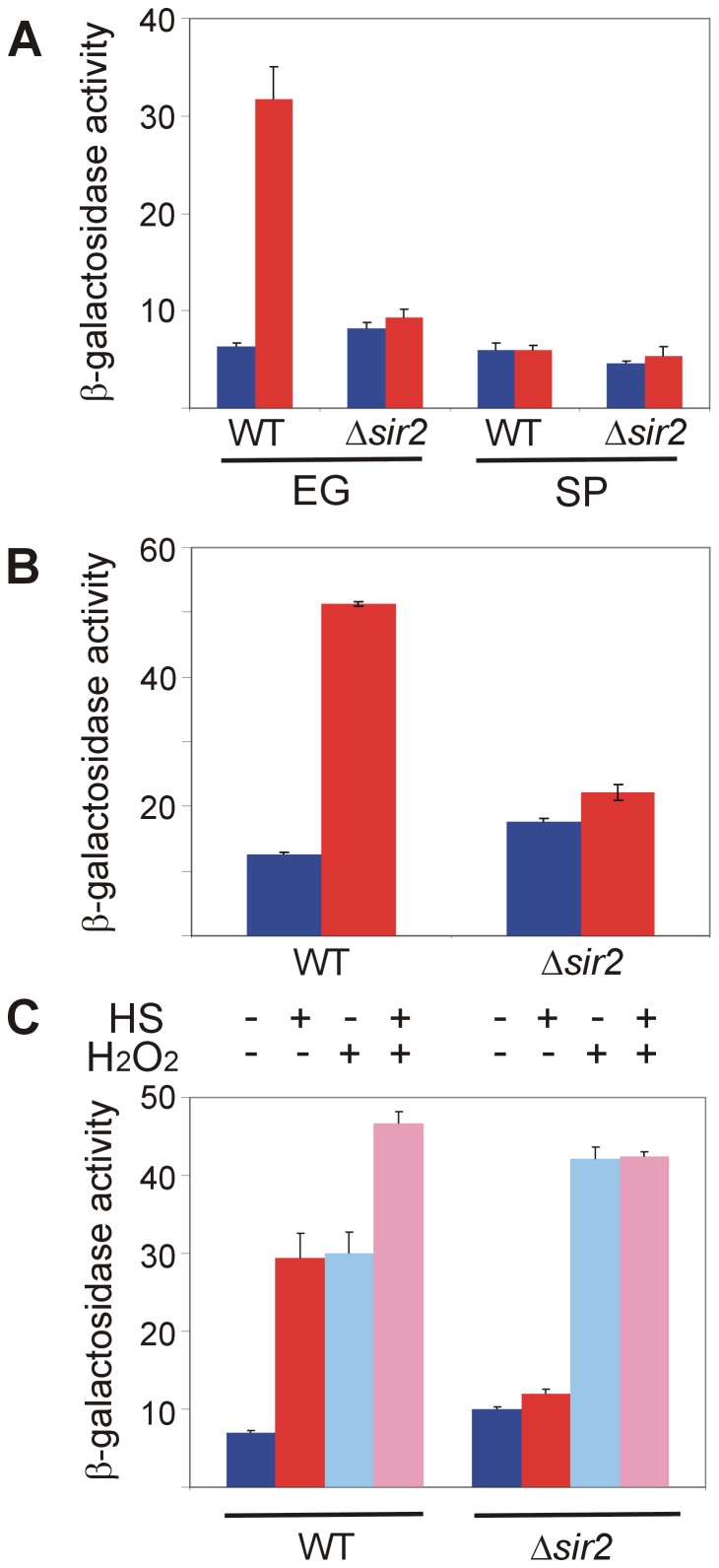
Sir2 is required for Hsf1 response to heat shock but not to oxidative stress. Wild-type and Δ*sir2* BY4741 cells (A), or wild-type and Δ*sir2* W303-1b cells (B), harboring HSE2-*LacZ* plasmid, were grown at 30°C either exponentially (EG) or to stationary-phase (SP). Cells were either incubated for 20 min at 30°C (blue bars) or subjected to 20 min heat shock at 42°C (red bars). (C) Exponentially growing wild-type and Δ*sir2* BY4741 cells harboring HSE2-*LacZ* plasmid were incubated for 30 min with (+) or without (−) H_2_O_2_ (3 mM) prior to the heat shock. Cells were either incubated further for 20 min at 30°C (−) or subjected to a 20 min heat shock (HS) at 42°C (+). Hsf1 activity was measured as β-galactosidase specific activity. The data are mean plus standard error of at least 3 independent experiments.

A role for NAD^+^ in aging makes sense in light of the involvement of sirtuins in lifespan determination. These class III protein deacetylases that consume NAD^+^ are implicated in lifespan extension in many model organisms and in particular in mediating the beneficial effects of dietary restriction [39]–[41]. Sir2, the founding member of the sirtuins family, exerts opposite effects on *S. cerevisiae* aging, depending on the yeast aging model system. While RLS is extended by excess *SIR2* and shortened upon *SIR2* deletion, CLS is prolonged in Δ*sir2* mutant under dietary restriction [4], [6], [43], [44]. Despite the enigmatic contribution of Sir2 to yeast aging, we examined whether activation of Hsf1 was modified in mutants lacking the *SIR2* gene. Following β-galactosidase activity, we found that although exponentially-growing Δ*sir2* cells (two different strains, BY4741 (Figure 6A) and W303-1b (Figure 6B)) exhibited somewhat higher basal Hsf1 activity than their wild-type counterparts, these mutants totally failed to respond to heat shock. In stationary-phase yeast, *SIR2* deletion had no effect on the residual basal or heat shock-induced activities of Hsf1 (Figure 6A).

Our data implicate Yap1 in the Hsf1 response to oxidative stress but not to heat shock (Figure 4), suggesting two distinct Hsf1 activation pathways. Therefore, it was interesting to determine whether Sir2 was restricted to the heat shock activation mode of Hsf1. Clearly, Δ*sir2* cells failed to respond to heat shock but maintained their full response to oxidative stress, similarly augmenting their β-galactosidase activity when exposed to H_2_O_2_ alone or in combination with heat shock (Figure 6C). This was in contrast to wild-type cells, which responded independently to either stress, and with augmented activity when both stressors were combined (Figure 6C; see also Figure 4). These findings exclude Sir2 from the activation of Hsf1 by oxidative stress and demonstrate that it functions in the heat shock activation pathway.

**Figure 7:**
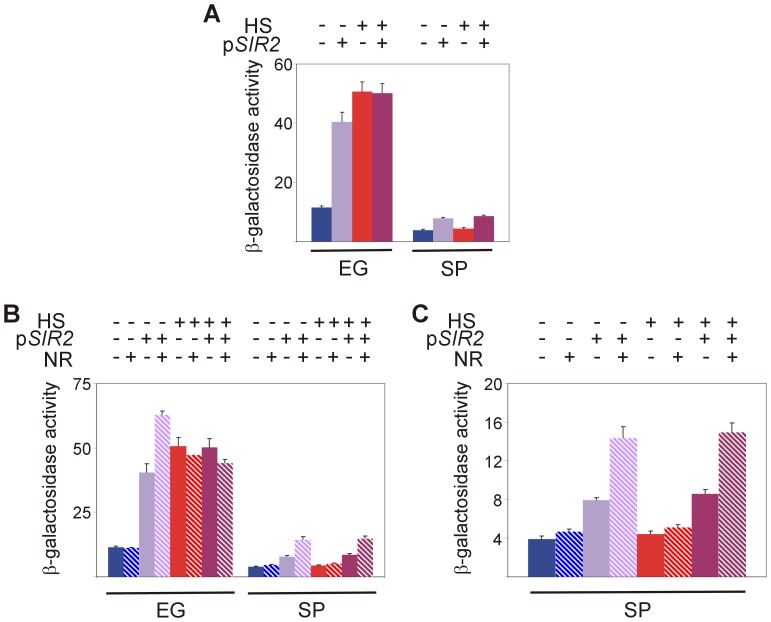
Activation of Hsf1 by heat shock is mimicked by excess Sir2 and improved by the NAD^+^ precursor. (A) Wild-type BY4741 cells harboring HSE2-*LacZ* plasmid were transformed with an empty vector (−) or a centromeric p*SIR2* plasmid (+). Cells grown at 30°C either exponentially (EG) or to stationary-phase (SP) were either incubated for 20 min at 30°C (−) or subjected to a 20 min HS at 42°C (+). (B) Wild-type BY4741 cells harboring HSE2-*LacZ* plasmid were transformed with an empty vector (−) or a p*SIR2* plasmid (+). Cells grown at 30°C to the indicated growth phase were incubated for 30 min with (+) or without (−) NR (10 µM) prior to the heat shock. Cells were either incubated further for 20 min at 30°C (−) or subjected to a 20 min heat shock (HS) at 42°C (+). (C) Activity in SP yeast from (B) drawn to a smaller scale. Hsf1 activity was measured as β-galactosidase specific activity. The data are mean plus standard error of at least 3 independent experiments.

To substantiate the role of Sir2 in Hsf1 activation by heat shock, we expressed in wild-type yeast excess *SIR2* from a plasmid. Clearly, in exponentially-growing yeast excess Sir2 mimicked the effect of heat shock and there was no further increase in Hsf1 activity by heat shock (Figures 7A and S3A). However, while the NAD^+^ precursor NR had no effect on Hsf1 activity in exponentially-growing naive yeast (Figures 7B and S3B; see also Figure 5), NR exerted increased Hsf1 activity in cells expressing excess *SIR2* (Figures 7B and S3B). We next tested the effect of excess *SIR2* and NR also in stationary-phase cells, and, again, while NR by itself had no effect (Figures 7B and S3B) and excess *SIR2* by itself increased Hsf1 activity by two-fold (Figures 7 and S3), supplementing stationary-phase yeast expressing excess *SIR2* with NR increased Hsf1 activity nearly four-fold, yet there was no additional effect of heat shock (Figures 7B,C and S3 B,C).

### Excess Hsf1, Sir2 and NAD^+^ precursor rejuvenate heat shock response in stationary-phase yeast

**Figure 8:**
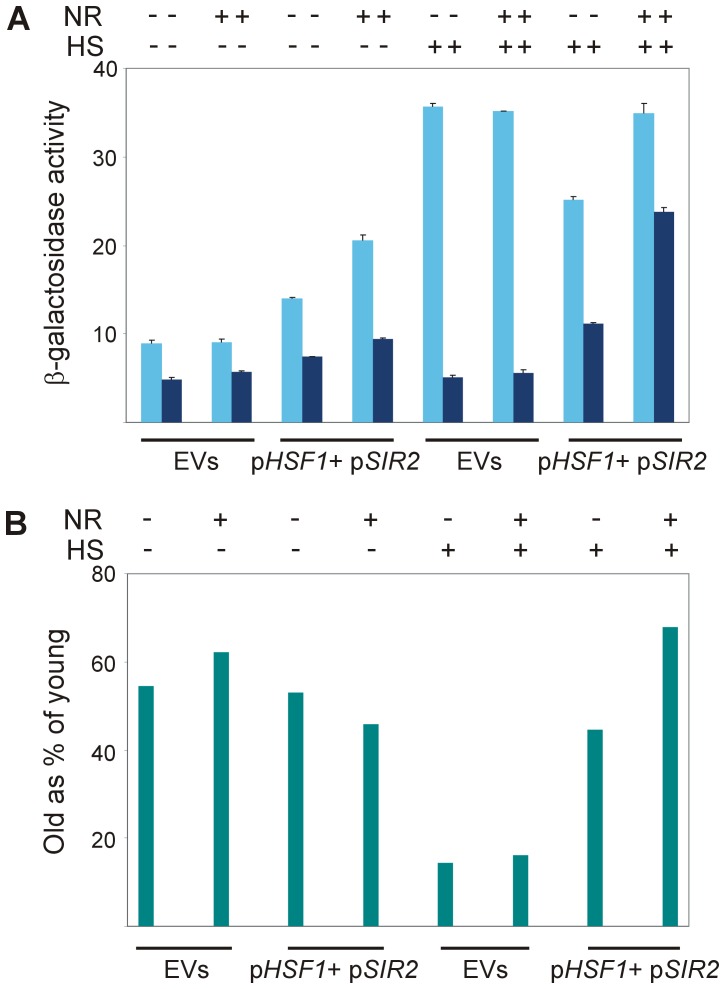
Activation of Hsf1 by heat shock is restored in stationary-phase yeast by combination of excess Hsf1, excess Sir2 and NAD^+^ precursor. (A) Wild-type W303-1b cells harboring HSE2-*LacZ* plasmid were transformed with empty vectors (EVs) or with a combination of p*HSF1*+p*SIR2* plasmids. Cells grown at 30°C, either exponentially (light blue bars) or to stationary-phase (dark blue bars), were incubated for 30 min with (+) or without (−) NR (10 µM) prior to the heat shock. Cells were either incubated further for 20 min at 30°C (−) or subjected to a 20 min heat shock (HS) at 42°C (+). Hsf1 activity was measured as β-galactosidase specific activity. Data are the mean plus standard error of 3 independent experiments. (B) At each treatment, the activity in stationary-phase cells was calculated as % of the activity in exponentially-growing cells.

Our findings in Figures 5–7 suggested that both Sir2 and NAD^+^ were limiting in stationary-phase yeast. Yet, additional factors seemed to be limiting in the Hsf1 activation cascade, as the heat shock response in these cells fell short of that in exponentially-growing yeast (Figures 7 and S3). Moreover, while the effect of excess Sir2 was further augmented by NR, neither in exponentially-growing nor in stationary-phase yeast was the excess Sir2 (with or without NR) further increased by heat shock (Figures 7 and S3). A plausible candidate for such a limiting factor was Hsf1 itself, since excess Hsf1 improved Hsf1 activity in stationary-phase yeast (Figure 2). Indeed, overexpressing Hsf1 together with Sir2 and providing the cells with NR restored the heat shock response of stationary-phase yeast to nearly 70% of that of exponentially-growing cells (Figure 8). Thus, limiting levels of three factors appear to impair the ability of stationary-phase yeast to respond to heat shock: Hsf1, Sir2 and NAD^+^.

## Discussion

**Figure 9:**
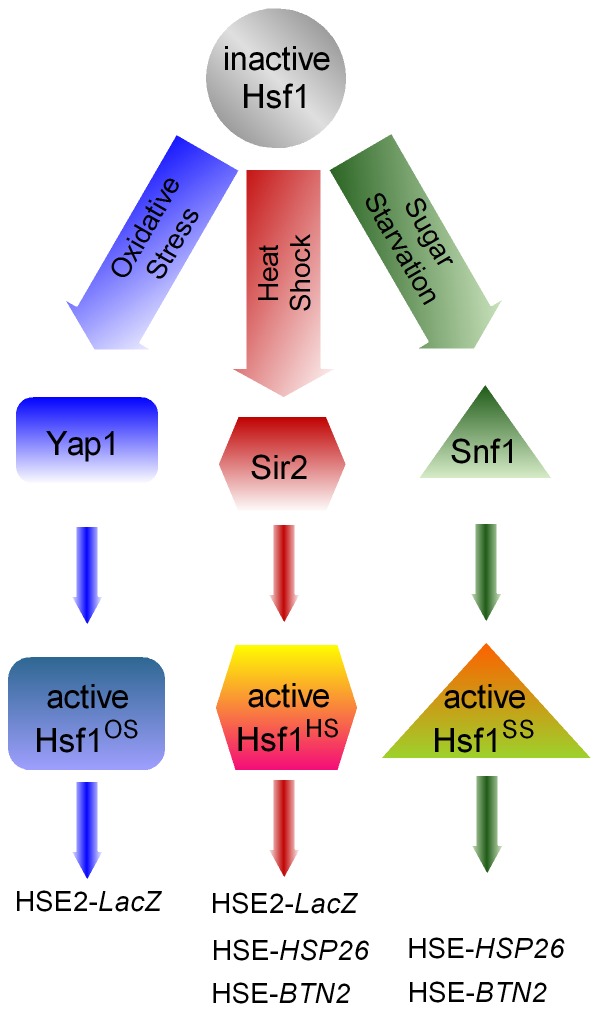
A schematic presentations of the various Hsf1 activation pathways. The three stresses, heat shock, oxidative stress and sugar starvation, activate the inactive Hsf1 through different mediators, Sir2, Yap1 and Snf1, respectively. Consequently, three distinct types of active Hsf1 are generated, HSF1^HS^, Hsf1^OS^ and Hsf1^SS^, respectively. These, in turn, transactivate the transcription of the indicated subsets of target genes.

The current studies establish Hsf1 as a longevity-related gene also in yeast, as its activation by heat shock or oxidative stress deteriorates in stationary-phase cells. We also provide evidence for two mediators of Hsf1 activation, Sir2 and Yap1, which operate in two discrete activation pathways: Sir2 in the heat shock response and Yap1 in the oxidative stress response (Figure 9). Our direct measurements of Hsf1 activity are based on three reporters with distinct HSEs, which respond differently to the three stressors tested. All three reporters respond to heat shock by increasing the levels of the proteins encoded by them. However, only HSE2-*lacZ*, driven by the synthetic HSE, is activated by oxidative stress yet it is indifferent to glucose starvation. Conversely, the genes driven by the perfect type endogenous HSEs, *HSP26* and *BTN2*, are activated by glucose starvation, but are indifferent to oxidative stress. This differential reaction to stress challenges emphasizes the specificity and modularity of the Hsf1 response, which is reflected by distinct subsets of responsive genes but more importantly, by unique modes of Hsf1 activation (Figure 9).

Clearly, Hsf1 response to either heat shock or oxidative stress declines progressively and is completely lost in stationary-phase yeast (Figure 1). Our findings in *S. cerevisiae*, showing that yeast Hsf1 is a longevity-related gene, echo studies in *C. elegans*, where Hsf1 has been shown to be essential for lifespan extension and to extend lifespan when overexpressed [37], [38]. Declined response to heat shock and oxidative stress has also been reported in old flies, aged rat tissues and senescent human cells [9], [46], [71], [72]. These effects on Hsf1 in stationary-phase yeast are neither due to considerable decline in Hsf1 levels nor to impaired ability to upregulate its target genes. This is indicated by the marginal increase in Hsf1 activity in stationary-phase yeast overexpressing *HSF1* (Figure 2) and by the activation of Hsf1 by glucose starvation that is maintained in stationary-phase yeast (Figure 3). To conclude, here we show that also in yeast, Hsf1 links responses to stress with lifespan, but it remains to be determined if the failure of Hsf1 to undergo activation is a cause or consequence of aging and whether lifespan extension requires Hsf1 and/or maintains activation-competent Hsf1.

The modularity of the Hsf1 activity is underscored by its three targets that respond differently to the three stresses elicited (Figure 9). Our model indicates that Hsf1 undergoes distinct modes of activation (denoted by different shapes and superscripts) by discrete and independent pathways. The different response of Hsf1 in stationary-phase yeast to the three stresses we employ indicates that specific factors operate in each stress pathway to mediate Hsf1 activation. Thus, the factor(s) involved in the response to glucose starvation appears to survive the transition from exponential growth to stationary-phase and is therefore distinct from factors that participate in the response to heat shock or oxidative stress, which are compromised during this transition. Indeed, Snf1 has been shown to be essential for Hsf1 activation by glucose starvation (as monitored by elevation of Hsp26 mRNA), but this kinase is not required for Hsf1 response to heat shock [35]. Significantly, although both responses decline in stationary-phase yeast, our data distinguish between the heat shock pathway and the oxidative stress pathway (Figure 9). The transcription factor Yap1 is implicated in the response of Hsf1 to oxidative stress but excluded from the heat shock response (Figure 4). Conversely, the NAD^+^-dependent Sir2 is implicated in Hsf1 response to heat shock but excluded from the oxidative stress (Figure 6).

Of particular interest is our finding that Hsf1 response to heat shock stringently depends on Sir2 (Figure 6) and is mimicked by excess Sir2 (Figures 7 and S3). The role of Sir2 in Hsf1 activation is also supported by the small but consistent increased heat shock response in stationary-phase yeast supplemented with NR or NAM (Figure 5). Indeed, NAM, unlike NR, was reported to be a noncompetitive inhibitor of Sir2 [73] and was shown by us to affect protein aggregation in a manner resembling *SIR2* deletion [45]. However, the similar effects on Hsf1 heat shock response exerted by NAM or NR (Figure 5) suggest that both compounds replenish intracellular NAD^+^, the levels of which are reported to decline in aging yeast and mice [65]–[68]. Furthermore, the effect of excess Sir2 is augmented in the presence of NR, and combination of excess Sir2 and NR partially restores Hsf1 activation also in stationary-phase yeast (Figures 7 and S3). Since sirtuins consume NAD^+^ in their deacetylation reaction, it is possible that their activity is regulated by cellular [NAD^+^]/[NADH] ratios, hence responds to changes in cellular metabolism, another hallmark of aging [74]. Such modulation of sirtuins' activity by the metabolic status of the cell adds yet another layer of regulation to Hsf1 functions in orchestrating stress responses. The clear involvement of Sir2 in the activation of yeast Hsf1 by heat shock (Figures 6–8 and S3) corresponds with similar findings regarding Sirt1 in mammals. It has been shown that Sirt1, the closest mammalian homolog of Sir2, deacetylates the mammalian Hsf1 as one of its activation modes [46]. By contrast, in *C. elegans* the heat shock response is independent of the Sir2/Sirt1 homologue Sir2.1, although the synergistic effect of dietary restriction and heat shock requires this sirtuin [47]. In our hands, the enhanced Hsf1 activation by the NAD^+^ precursor NR, which is further augmented when excess Sir2 is expressed, strongly suggests that yeast Sir2 acts in the heat shock response as Hsf1 deacetylase, as does Sirt1 in mammals.

To conclude, although much about cellular aging in yeast and in general remains obscure, the current work unveils some of the players and pathways that affect and are affected by aging. Our data indicate that at least three components in the Hsf1 heat shock activation pathway are limiting in stationary-phase yeast, Hsf1 itself, Sir2 and its cofactor NAD^+^. When supplemented in combination, excess *HSF1*, excess *SIR2* and the NAD^+^ precursor NR can rejuvenate to a large extent the heat shock response in stationary-phase yeast (Figure 8). If restoring the heat shock response also slows down aging, it would indicate that its decline is a cause rather than a consequence of aging. Finally, the aging-dependent changes in Hsf1 response described here, combined with the effects of aging on the aggregation of a polyglutamine-containing protein we previously reported [45], establish *S. cerevisiae* as a suitable model organism not merely for lifespan studies ending in cell death, but also for research addressing various molecular aspects of the aging process.

## Supporting Information

Figure S1 **Hsf1 activation by sugar starvation is maintained in stationary-phase yeast but poorly reported by HSE2-** ***LacZ*** **.** Exponential BY4741 cells expressing Hsp26-GFP were grown at 30°C in SC medium containing 2% (w/v) galactose (A), and exponential BY4741 cells harboring HSE2-*LacZ* plasmid were grown at 30°C in SC medium containing 2% (w/v) glucose (B). Cells were transferred to fresh media supplemented with the standard 2% or low 0.05% sugar and were either maintained at exponential growth (EG) or allowed to reach stationary-phase (SP) in these media. Prior to heat shock, cells were transferred to fresh media supplemented with the respective 2% or 0.05% sugar and further incubated at 30°C for 3 hrs. Cells were either incubated for 20 min at 30°C (−) or subjected to a 20 min HS at 42°C (+). Hsf1 activity was measured as (A) levels of Hsp26-GFP relative to actin (a loading control), as determined by quantified immunoblotting or (B) β-galactosidase specific activity. The data are the mean of 2–3 independent experiments. Similar Hsp26-GFP levels were obtained in cells grown in either galactose or glucose.
(TIF)

Figure S2 **Hsf1 response to oxidative stress is poorly reported by Hsp26-GFP or Btn2-GFP.** BY4741 cells expressing Hsp26-GFP (A) or Btn2-GFP (B) grown at 30°C either exponentially (EG) or to stationary-phase (SP) were incubated for 30 min with (+) or without (−) H_2_O_2_ (3 mM) prior to heat shock. Cells were either incubated further for 20 min at 30°C (−) or subjected to a 20 min to heat shock (HS) at 42°C (+). Hsf1 activity was measured as levels of Hsp26-GFP (A) or Btn2-GFP (B) relative to actin (a loading control), as determined by quantified immunoblotting. The data are the mean plus standard error of at least 5 independent experiments.
(TIF)

Figure S3 **Activation of Hsf1 by heat shock is mimicked by excess Sir2 and improved by the NAD^+^ precursor.** (A) Wild-type W303-1b cells harboring HSE2-*LacZ* plasmid were transformed with an empty vector (−) or a centromeric p*SIR2* plasmid (+). Cells grown at 30°C either exponentially (EG) or to stationary-phase (SP) were either incubated for 20 min at 30°C (−) or subjected to a 20 min HS at 42°C (+). (B) Wild-type W302-1b cells harboring HSE2-*LacZ* plasmid were transformed with an empty vector (−) or a p*SIR2* plasmid (+). Cells grown at 30°C to the indicated growth phase were incubated for 30 min with (+) or without (−) NR (10 µM) prior to the heat shock. Cells were either incubated further for 20 min at 30°C (−) or subjected to a 20 min heat shock (HS) at 42°C (+). (C) Activity in SP yeast from (B) drawn to a smaller scale. Hsf1 activity was measured as β-galactosidase specific activity. The data are mean plus standard error of at least 3 independent experiments.
(TIF)
