# Supplementary figures and images for: Deteriorated Stress Response in Stationary-Phase Yeast: Sir2 and Yap1 Are Essential for Hsf1 Activation by Heat Shock and Oxidative Stress, Respectively
Source: PLoS One. 2014 Oct 30;9(10):e111505. doi: 10.1371/journal.pone.0111505 (PMC4214751; doi:10.1371/journal.pone.0111505)

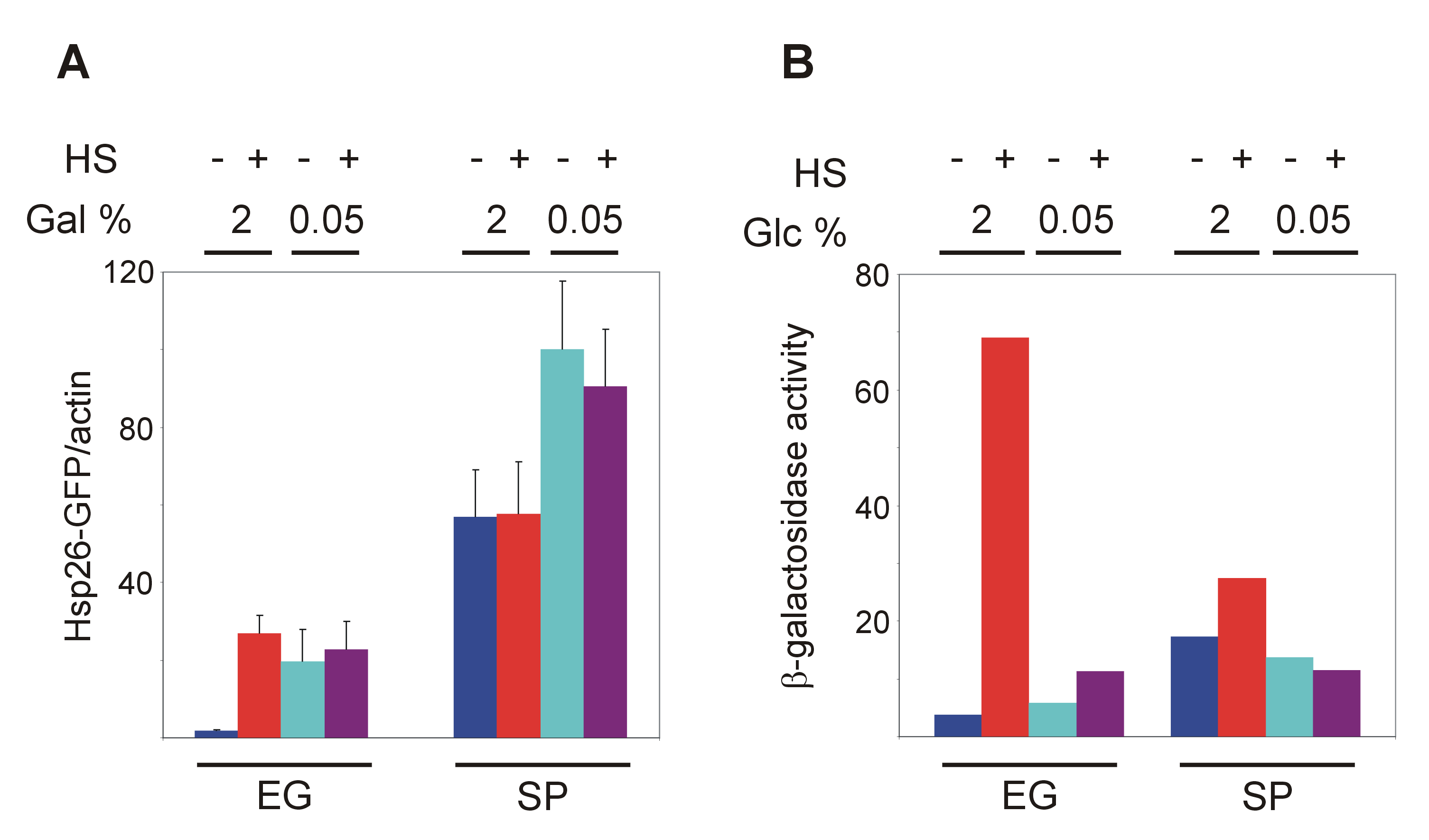

Supplement: Figure S1 — Hsf1 activation by sugar starvation is maintained in stationary-phase yeast but poorly reported by HSE2- LacZ . Exponential BY4741 cells expressing Hsp26-GFP were grown at 30°C in SC medium containing 2% (w/v) galactose (A), and exponential BY4741 cells harboring HSE2-LacZ plasmid were grown at 30°C in SC medium containing 2% (w/v) glucose (B). Cells were transferred to fresh media supplemented with the standard 2% or low 0.05% sugar and were either maintained at exponential growth (EG) or allowed to reach stationary-phase (SP) in these media. Prior to heat shock, cells were transferred to fresh media supplemented with the respective 2% or 0.05% sugar and further incubated at 30°C for 3 hrs. Cells were either incubated for 20 min at 30°C (−) or subjected to a 20 min HS at 42°C (+). Hsf1 activity was measured as (A) levels of Hsp26-GFP relative to actin (a loading control), as determined by quantified immunoblotting or (B) β-galactosidase specific activity. The data are the mean of 2–3 independent experiments. Similar Hsp26-GFP levels were obtained in cells grown in either galactose or glucose. (TIF) [file pone.0111505.s001.tif]

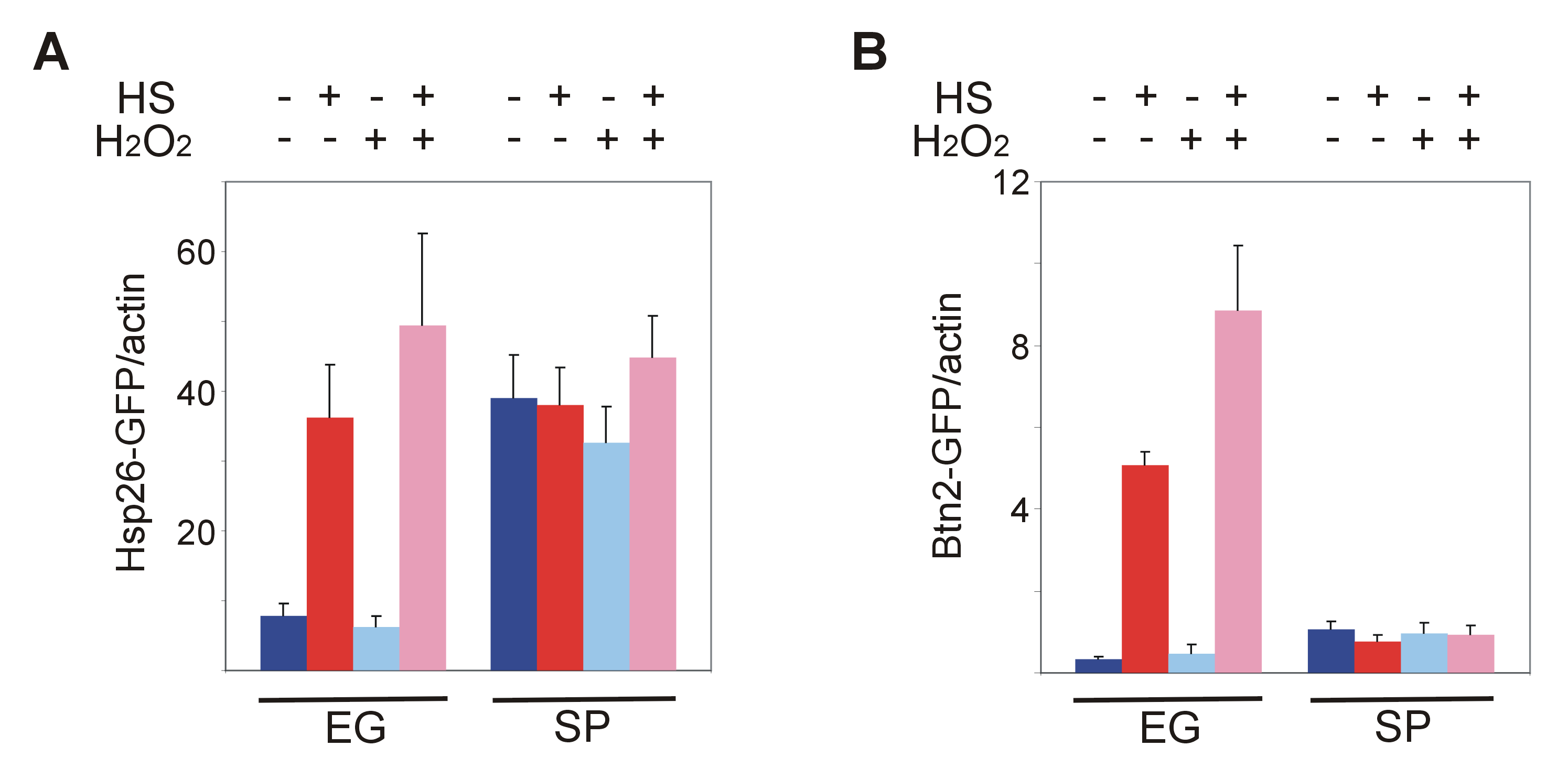

Supplement: Figure S2 — Hsf1 response to oxidative stress is poorly reported by Hsp26-GFP or Btn2-GFP. BY4741 cells expressing Hsp26-GFP (A) or Btn2-GFP (B) grown at 30°C either exponentially (EG) or to stationary-phase (SP) were incubated for 30 min with (+) or without (−) H2O2 (3 mM) prior to heat shock. Cells were either incubated further for 20 min at 30°C (−) or subjected to a 20 min to heat shock (HS) at 42°C (+). Hsf1 activity was measured as levels of Hsp26-GFP (A) or Btn2-GFP (B) relative to actin (a loading control), as determined by quantified immunoblotting. The data are the mean plus standard error of at least 5 independent experiments. (TIF) [file pone.0111505.s002.tif]

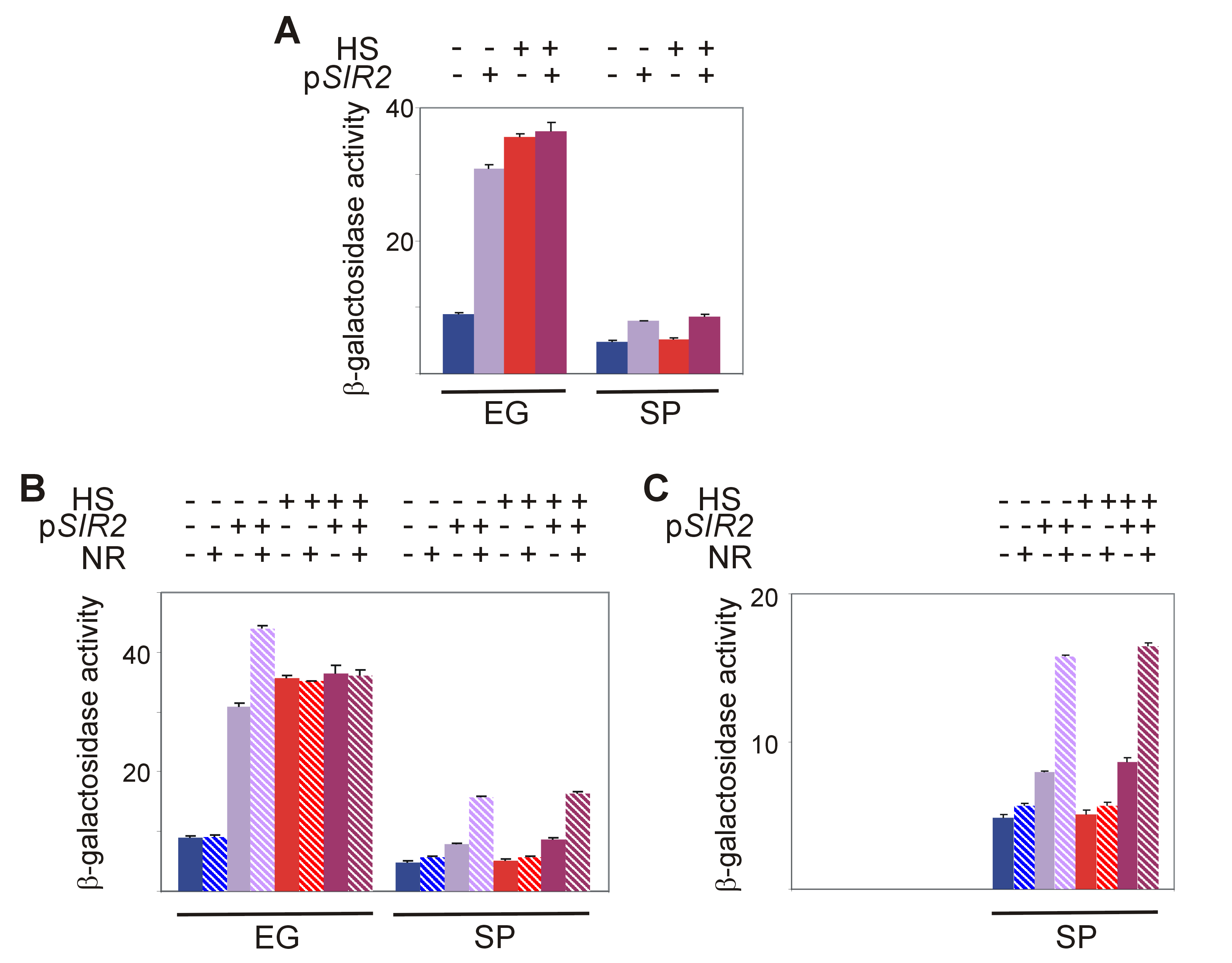

Supplement: Figure S3 — Activation of Hsf1 by heat shock is mimicked by excess Sir2 and improved by the NAD+ precursor. (A) Wild-type W303-1b cells harboring HSE2-LacZ plasmid were transformed with an empty vector (−) or a centromeric pSIR2 plasmid (+). Cells grown at 30°C either exponentially (EG) or to stationary-phase (SP) were either incubated for 20 min at 30°C (−) or subjected to a 20 min HS at 42°C (+). (B) Wild-type W302-1b cells harboring HSE2-LacZ plasmid were transformed with an empty vector (−) or a pSIR2 plasmid (+). Cells grown at 30°C to the indicated growth phase were incubated for 30 min with (+) or without (−) NR (10 µM) prior to the heat shock. Cells were either incubated further for 20 min at 30°C (−) or subjected to a 20 min heat shock (HS) at 42°C (+). (C) Activity in SP yeast from (B) drawn to a smaller scale. Hsf1 activity was measured as β-galactosidase specific activity. The data are mean plus standard error of at least 3 independent experiments. (TIF) [file pone.0111505.s003.tif]
